# A New Cure for Stuttering

**Published:** 1888-08

**Authors:** 


A NEW CURE FOR STUTTERING.—Coen (*Ctrbl. f. klin. Med.—Lyon. Med.*) acting on the principal that a stutterer does not stutter when he speaks in a low tone, advises the following course of treatment: Absolute silence for a preparatory period of eight or ten days; speaking only in a low tone for another period of the same number of days; and a gradual elevation of the voice during the next ten or fifteen days.—*Am. Med. Digest.*

